# In vitro antibacterial and cytotoxic effects of *Euphorbia grandicornis* Blanc chemical constituents

**DOI:** 10.1186/s12906-022-03571-8

**Published:** 2022-03-25

**Authors:** Douglas Kemboi, Moses K. Langat, Xavier Siwe-Noundou, Rui W. M. Krause, Michelle Louise Isaacs, Vuyelwa Jacqueline Tembu

**Affiliations:** 1 Department of Chemistry, Tshwane University of Technology, Pretoria, 0001 South Africa; 2 Department of Chemistry, Rhodes University, Makhanda (Grahamstown), 6140 South Africa; 3 Royal Botanic Gardens Kew, Kew Green, Richmond, Surrey, TW9 3AE UK; 4 Pharmaceutical Sciences Department, School of Pharmacy, Sefako Makgatho Health Sciences University, Pretoria, 0204 South Africa; 5 Department of Biochemistry and Microbiology, Rhodes University, Makhanda (Grahamstown),, 6140 South Africa

**Keywords:** *Euphorbia grandicornis*, Chemical constituents, *Staphylococcus aureus* ATCC 6538 and *Escherichia coli* ATCC 8739

## Abstract

**Background:**

*Euphorbia grandicornis* is widely utilized in traditional medicine for the treatment of microbial infections including sexually transmitted diseases such as syphilis, gonorrhoea and for healing of wounds*.*

**Objective:**

The aim of this work was to isolate and evaluate the antibacterial and anticancer activities of *Euphorbia grandicornis* chemical constituents.

**Methods:**

Chemical constituents were isolated and identified using various spectroscopic techniques such as IR, MS, and NMR. The single point growth inhibitory potential of the compounds was determined using a 96-well plate based assay.

**Results:**

The CH_2_Cl_2_ crude extracts exhibited potent antibacterial activity against *Escherichia coli* ATCC 8739 and *Staphylococcus aureus* ATCC 6538 with percentage growth of 94.90 ± 4.24 and 29.47 ± 4.89 respectively*.* Hence, the CH_2_Cl_2_ crude extract was further subjected to column chromatography which resulted in the isolation of methyl 2,5-dihydroxybenzoate (**1**), *n*-octyl benzoate (**2**), friedelanol (**3**), and germanicol (**4**) and identification of compounds 12–24 for the first time in the species based on the LC-MS/MS spectroscopic data. The purified compounds (**1**–**4**), and previously reported compounds (**5**–**11**) were evaluated for antibacterial activities against *S. aureus* and *E. coli,* as well as the cytotoxicity effects against HeLa cells. Of the purified compounds, methyl 2,5-dihydroxybenzoate (**1**), was the most active against *E.coli* and *S. aureus* with a percentage growth of 19.12 ± 0.65 and 23.32 ± 0.23 respectively. *β*-amyrin (**6**), and *β*-sitosterol (**8**), were active against *S. aureus* with percentage growth of 27.17 ± 0.07, and 47.79 ± 2.99 respectively.

**Conclusion:**

The results obtained from this study indicate that *E. grandicornis,* is a rich source of chemical constituents that may provide new lead compounds for the development of antibacterial agents.

**Supplementary Information:**

The online version contains supplementary material available at 10.1186/s12906-022-03571-8.

## Introduction

The rising cases of antibiotic resistance and shortage of bioactive antimicrobial drugs remain a major threat to global health [1, 2]. The resistance of pathogens to commercially available drugs possess a major challenge to the attainment of universal health as envisaged in the sustainable development goals on the heath [3, 4]. Bacterial strains like *Staphylococcus aureus* and *Escherichia coli* are involved in the many respiratory and skin infections, which cause urogenital, gastrointestinal, and wound contamination, are some of the resistant strains to most of the available antibiotics [5, 6]. Furthermore, the antibiotics commonly used are sometimes associated with adverse side effects such as hypersensitivity, allergic reactions and immune suppression, which makes treatment of bacterial infections difficult [6].

Therefore, there is a need to discover novel therapeutics utilizing naturally occurring lead compounds or pharmacophores from medicinal plants, which can be used to synthesize new antibacterial drugs that are more effective and less toxic. This is because medicinal plants are readily available and are known to synthesize bioactive compounds. In addition, the use of non-toxic natural therapies has achieved success over the past few decades [7]. It is also estimated that 40–60% of the population in developing countries continue to use medicinal plants as complementary medicine in the treatment of infectious and chronic diseases [8]. Medicinal plants used as complementary medicine also showed significant pharmacological properties [9]. Notwithstanding, an ideal chemo-preventive or therapeutic agent for humans should have little or no toxicity, anti-mutagenic, anti-carcinogenic activities; for human cells [9, 10], a known mechanism of actions, the capability of consumption orally, readily available, affordable, and human acceptance. All which plants and their constituents have proved to possess. Therefore, plants could provide lead compounds for drug discovery, particularly as antibacterial agents [10].

The wide distribution of *Euphorbia* species in Africa and their different forms of growth have attracted human interest since ancient times and are utilized in the treatment of several illnesses [11–13]. Reported literature shows that most species are used in traditional medicine for the treatment of diseases ranging from microbial and respiratory infections, body and skin irritations, digestion complaints, inflammation, body pain, snake and scorpion bites, nutritional disorders to endocrine disorders and pregnancy or birth disorders [11, 12, 14, 15]. Many records described the use of *Euphorbia* species for the treatment of microbial infections including sexually transmitted diseases such as syphilis and gonorrhoea. Other medicinal uses include tuberculosis, ringworms, malaria, and anthelmintic [11, 12].

The extracts of these species were reported to possess antibacterial, anti-inflammatory, antioxidant, antitumor, antispasmodic, antidiabetic, ant-eczema, antitussive, and anti-proliferative properties [12, 13]. The diverse medicinal uses of *Euphorbia* species are often related to the presence of bioactive compounds like macrocyclic and polycyclic diterpenoids. For instance, *ent*-abietane diterpenoids isolated from roots extracts of *E. wallichii* were evaluated for antimicrobial activity against six Gram-positive bacteria including *Eorynebacterium* (T25–17), *Enterococcus* species (8152), *Enterococcus faecalis* (C159–6), and Gram-negative bacteria including *Citrobacter freundii* (11041), *Acinetobacter baumanii* (9010) and *A. baumanii* (9011) using gentamicin as a positive control. 11*β*-hydroxy-14-oxo-17-al-ent-abieta-8(9),13(15)-dien-16,12*β*-olide,11*β*,17-dihydroxy-12-methoxy-ent-abieta-8(14), 13(15)-dien-16,12A-olide and 14A-hydroxy-17-al-entabieta-7(8),11(12), 13(15)-trien-16,12-olide were found to exhibit significant antimicrobial activities against the three Gram-positive bacteria with MIC value of 60.00 μg/mL but they were inactive against the Gram-negative bacteria as compared to gentamicin as the control [16]. However, among the *Euphorbia* species, *Euphorbia grandicornis* Blanc is one of the unexplored species that is native to the African continent where it is cultivated for its use in the treatment of antimicrobial infections and wound healing [17]. The current research was therefore aimed at investigating the antibacterial constituents of *Euphorbia grandicornis* Blanc.

## Materials and methods

### General

Column chromatography was performed on polyamide columns (5 × 60 cm, 200 g) (Germany GmbH) over silica gel (Kieselgel 60 GF_254_, 15 μm, Merck, Germany). While Thin Layer Chromatography (TLC) was carried out on Kieselgel 60 F_254_ (Merck). Spots on UV active silica gel were detected under UV light (245 and 336 nm) and made visible using a concentrated sulphuric-anisaldehyde spray mixture and heated at 105 °C for 2 min.

All the NMR experiments were performed on a Bruker Avance spectrometer (Bruker spin AG, DRX, Switzerland 400.13 MHz; 100.62 MHz). All spectral data were referenced to solvent peaks (CDCl_3_, δ_H_: 7.25, δ_C_: 77.24 ppm recorded at 25 °C. Melting points was determined on an Ernst LeitzWetziar micro-hot stage melting point apparatus and were reported uncorrected. IR spectra were measured on PerkinElmer spectrometer, version 10.54 and the absorptions were recorded in wavenumbers (cm ^− 1^).

The specific optical rotation was performed using Jasco P-2000 polarimeter (JASCO, Germany) using a 10 cm length. The High-Resolution LC-MS analysis was performed on a Bruker Daltonics Compact QTOF Mass Spectrometer in the positive mode. The 5 min run was achieved using an Acclaim RSLC 120 C18, 2.2 μm, 2.1 × 100 mm (P/N 068982) column at 40 °C, flow rate 0.2 ml/min. The solvent system used was Water-Acetonitrile (10:90, v/v) each solvent containing 0.1% of formic acid [18, 19].

The liquid chromatography-mass spectrometry (LC-MS), with a Waters Synapt G2 quadrupole time-of-flight (QTOF) mass spectrometer (MS), connected to a water ultra-performance liquid chromatography (UPLC) (Waters, Milford, MA, USA) was used for the analysis of the crude extracts. Electrospray ionization was used in negative mode with a cone voltage of 15 V, desolvation temperature of 275 °C, gas at 650 L/h, and the rest of the MS settings optimized for best resolution and sensitivity. Data were acquired by scanning from m/z 150 to 1500 m/z in resolution mode as well as in MSE mode. In MSE mode two channels of MS data were acquired, one at low collision energy (4 V) and the second using a collision energy ramp (40–100 V) to obtain fragmentation data as well. Leucine enkaphalin was used as lock mass (reference mass) for accurate mass determination and the instrument was calibrated with sodium formate [18]. The analysis was done at Stellenbosch University, LC-MS unit.

### Plant material

The roots and aerial parts of *Euphorbia grandicomita* plants were collected in summer at the Limpopo province, Wild flowers nursery, South Africa, S 05°04.579′ E 043°35.035′, in November 2017. The plant specimen was identified by Erich Van Wyk, at the South African National Biodiversity Institute (SANBI), Pretoria, South Africa and the specimen was deposited with a voucher number of 18,044 [19].

### Extraction and isolation

The fresh aerial (969.72 g) and roots (800.34 g) of *E. grandicomita* were chopped, dried, and separately percolated with dichloromethane (CH_2_Cl_2_) (2.5 L) at room temperature for 2 weeks. The CH_2_Cl_2_ extract was then concentrated under reduced pressure on a rotary evaporator to obtain the crude extracts. Fractionation of the root extract (23.5 g) resulted in 200 fractions of eluents, collected in 250 mL aliquots which were subsequently concentrated and subjected to TLC analysis. The TLC profiling of the aliquots resulted in the combination of fractions 1–49 (200.22 mg) eluted with 100% n-hexane as a fraction (**FO**). Similarly, TLC analysis of fractions 50–100 (fractions; 50–60 (n-hexane: CH_2_Cl_2_; 9:1 v/v) and 61–100; (n-hexane: CH_2_Cl_2_; 8:2 v/v) showed four spots of retention factor (Rf) values of 0.53 (major component), 0.52, 0.45 and 0.58, which turned purple on staining with anisaldehyde-conc.H_2_SO_4_ mixture and subsequent heating at 100 °C. The fractions were combined as **FA** (200.45 mg) and was further purified (n-hexane: CH_2_Cl_2_; 9: 0.5 v/v) to yield white crystals of friedelanol (**3**) (Rf = 0.53, 7.24 mg) which was collected as sub fractions 1–30. Sub fractions 51–70 were also combined and further purified using n-hexane: CH_2_Cl_2_; 8:2 v/v, to yield a white powder of germanicol (**4**) (Rf = 0.42, 10.32 mg). The mother liquor and sub-fractions 85–92 were combined and evaporated under reduced pressure on a rotary evaporator. Preparative TLC chromatography of this fraction using (n-hexane: EtOAc; 98:2 v/v) afforded white powder of *n*-octyl benzoate (**2**), (13.42 mg, Rf = 0.42). Furthermore, fractions 101–180 (Fractions 101–120 (n-hexane: CH_2_Cl_2_; 7:3 v/v) and fractions 121–180 (n-hexane: CH_2_Cl_2_; 2:8 v/v) had two main compounds having Rf values of 0.34 and 0.35 together with other minor compounds when developed in n-hexane: CH_2_Cl_2_; 1:1 v/v solvent mixture. The fractions were pooled together to form fraction **FB** (180.43 mg). Column chromatography of fraction FB gave 100 subfractions of 20 mL each. TLC analysis led to the combination of subfractions 1–50 which was dried out to give white powder of methyl 2,5-dihydroxybenzoate (**1**), (n-hexane:EtOAc; 3:7 v/v, Rf = 0.58, 8.12 mg).

### Antibacterial activities and cytotoxicity

The single point growth inhibitory potential of the samples was determined using a 96-well plate based assay. The bacterial strains used in this assay included *Escherichia coli* (ATCC 25922), and *Staphylococcus aureus* (ATCC 25923). HeLa cells were used to evaluate the cytotoxicity of the compounds. Overnight cultures of bacterial strains were maintained on Luria Agar plates streaked to single colonies and stored at 4 °C and were grown on liquid cultures in 5 mL Luria Broth test tubes. A single colony was selected using a sterile toothpick and were suspended in 5 mL LB medium and incubated on a shaker at 160 rpm overnight at 37 °C. Thereafter, 50 μL of overnight bacterial suspension was aspirated into a 5 mL of sterile LBM and was grown to log phase on a shaker (160 rpm) at 37 °C. Samples were reconstituted in dimethyl sulfoxide (DMSO) to a stock concentration of 20 mg/mL, and tested at a final concentration of 50 μg/mL. About 20 μL of 0.5 mg/mL of the extract and 20 μL bacterial suspension (1:100 *E. coli*; 1:10 *S. aureus*) were made to a final volume of 200 μL by addition of 160 μL LBM and the plates were incubated at 37 °C for 6 h in sealed plastic to avoid evaporation. Negative control 0% media only, no cells, and positive control 100% cells plus media (no extract) were used. To assess the percentage of cell viability, a resazurin viability reagent (20 μL of resazurin) was used. After the plates were scored visually, 20 μL of the prepared viability reagent was added to each well and the plates were incubated for an additional 20–60 min. This was followed by measuring the mean fluorescence intensity on a PerkinElmer Enspire microplate reader with the excitation/emission wavelengths set to 560/590 nm. The % bacterial growth was calculated using the following equation:

$$\%\mathrm{Bacterial}\ \mathrm{growth}=\frac{\mathrm{Sample}\ \mathrm{FI}-\Sigma \mathrm{blank}}{\Sigma \mathrm{negative}\ \mathrm{control}-\Sigma \mathrm{blank}} \times 100$$

To assess the cytotoxicity, compounds were incubated at a fixed concentration of 50 μg/mL in 96-well plates containing HeLa cells for 24 h. The numbers of cells surviving drug exposure were determined by using the resazurin-based reagent and reading resorufin fluorescence in a multiwell plate reader in compound-treated wells relative to untreated controls. The results were expressed as % cell viability, based on fluorescence reading in treated wells versus the untreated control well and measurements were done in triplicate.

### Isolated compounds

**Fig. 1:**
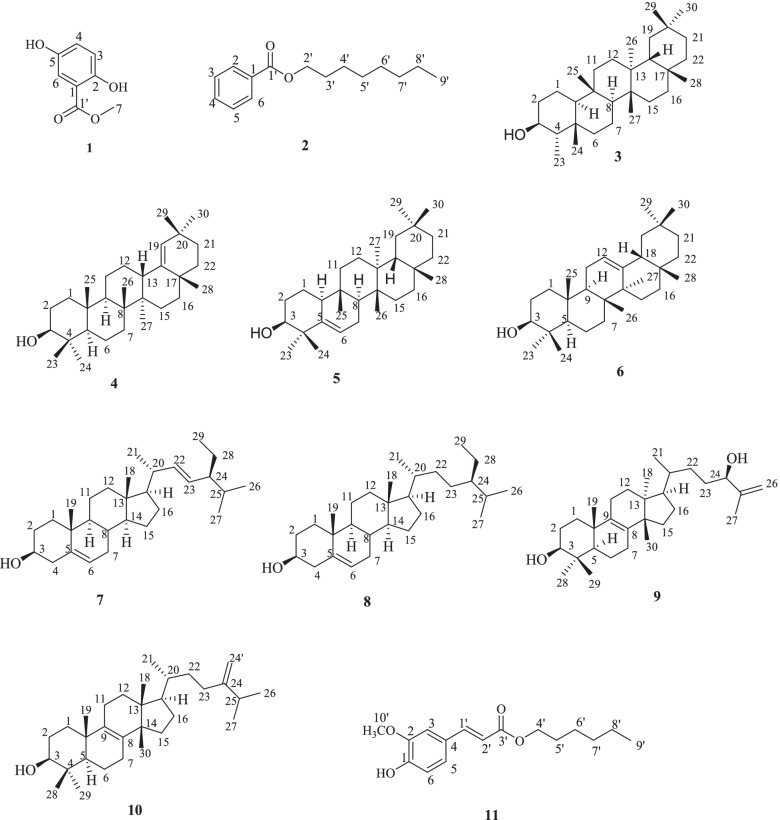
Chemical structures of isolated compounds from *E. grandicornis Blanc*

Repeated column chromatography and preparative thin-layer chromatography of *E. grandicornis Blanc* root extract afforded methyl 2,5-dihydroxybenzoate (**1**), *n*-octyl benzoate (**2**), *β*-friedelanol (**3**), and germanicol (**4**) for the first time in the species alongside (**5–11**) previously reported compounds [19] (Fig. 1).

Methyl 2,5-dihydroxybenzoate (1) was obtained from the CH_2_Cl_2_ extract of *E. grandicornis* as a white powder. The melting point was determined to be between 78 and 89 °C with its specific optical rotation recorded as [*α*]_D_^25^–75.53 (CHCl_3_, conc = 6.60 (w/v%) at 20 °C. The HR-ESI-MS exhibited a pseudo-molecular ion [M + H]^+^ at *m/z* 169.0107 which indicated a molecular formula C_8_H_8_O_4_ calculated for *m/z* 168.0423. The IR spectrum displayed absorptions bands at 2957.8 cm^− 1^ which revealed the presence of C = C and absorptions at 1214 cm^− 1^ due to C-O stretches of the methoxy group. Broad absorption bands at 3244 cm^− 1^ were attributed to the hydroxyl groups (OH). The ^1^H NMR (400 MHz) displayed a characteristic singlet peak of a methoxy with proton resonance at *δ*_H_ 3.94 (*s*, H-7) ppm, integrating to three protons. The spectrum also displayed aromatic protons at *δ*_H_ 6.93 (*d*, *J* = 12.0, H-5) and 7.29 7.41 (*d*, *J* = 1.7, H-3, H-6) ppm. The ^13^C NMR spectrum showed eight carbon resonances at *δ*_C_ 122.9 (C-1), 156.7 (C-2), 114.8 (C-3), and 118.5 (C-4) ppm. The spectrum also displayed a carbonyl resonance at *δ*_C_ 167.4 and a methoxy carbon resonance at *δ*_C_ 52.4 ppm. The NMR spectral data compared well with a known compound named; methyl 2,5-dihydroxybenzoate, previously isolated from *Illicium dunnianum* as in Tables S1 and S2 [20].

***n-*** Octylbenzoate, (2) was obtained from the CH_2_Cl_2_ extracts of *E. grandicornis* as a pure white powder. The melting point was determined between 75 and 95 °C with its optical specific rotation recorded as [*α*]_D_ + 54.10 (CHCl_3_, conc = 3.6 (w/v%) at 25 °C. The HR-ESI-MS spectrum showed a pseudo-molecular ion [M + H]^+^ at *m/z* 235.0723, calculated for C_15_H_22_O_2_, *m/z* 234.1620. The fragmentation patterns were consistent with that of a monosubstituted benzoate ester with a molecular peak at [M + H]^+^ *=* 116.1248 (C_8_H_17_)^+^ due to an octyl side-chain molecular ion, and at [M-H]^+^ *m/z* 123.0574 (C_7_H_6_O_2_)^+^ due to benzoic acid molecular ion. The IR spectrum exhibited an absorptions band at 1718 cm^− 1^ which revealed the presence of a carbonyl group, absorption band at 1107 cm^− 1^ due to C-O stretches and more absorption bands at 2913 cm^− 1^ and 2860 cm^− 1^ attributed to C-H (alky) stretches.

The ^1^H NMR (400 MHz) displayed characteristic signals of a benzoate ester with proton resonances at *δ*_H_ 8.02 (*d*, *J* = 7.2, H-2, H-6), 7.52 (*t*, *J* = 7.6, H-4, H-5) and 7.44 (*d*, *J* = 6.4, H-3) ppm. The spectrum exhibited a triplet proton resonance at *δ*_H_ 4.31 (*t*, *J* = 6.8) ppm. In addition, an upfield triplet proton resonance at *δ*_H_ 0.83 (*t, J =* 6.8, H-9′) ppm was attributed to the terminal methyl group, while a broad clustered proton resonance at *δ*_H_ 1.22 ppm accounted for twelve protons revealing the presence of six methylene groups. The ^13^C NMR spectrum displayed carbon resonances typical of a benzoate ester (*δ*_C_; 128.3, 129.5, 130.5, 132.7) [21], however, attached with an aliphatic side chain. The NMR spectral data for compound (2) were consistent with the synthetically reported *n-*octyl benzoate as in Tables S1 and S2 [22].

3 ***β*** -friedelanol, (3) was isolated as a white crystal. The melting point was determined to be between 192 and 197 °C while its optical rotation was determined to be [*α*]_D_^25^ + 34.20 (CHCl_3_, conc = 0.36 (w/v%). The HR-ESI-MS spectrum for this compound displayed a pseudo-molecular ion [M + H]^+^ at *m/z* 429.1763 indicating a molecular formula C_30_H_52_O calculated for *m/z* 428.7333. The IR spectrum exhibited absorption bands at 3340 cm^− 1^ due to the hydroxyl group and bands at 2925 and 2866 cm^− 1^ were ascribed to C-H stretches. Analysis of the ^1^H NMR (400 MHz) spectra displayed resonances due to an oxymethine proton at *δ*_H_ 3.66 (*t*, *J* = 1.9, 1H) ppm which correlated with a carbon resonance at *δ*_C_ 72.8 (C-3) ppm as observed in the HSQC spectrum. Other observed methyl proton resonances were *δ*_H_ 0.97 (*s*, 3H-23), 1.11 (*s*, 3H-24), 0.93 (*s*, 3H-25), 0.97 (*s*, 3H-26), 1.01 (*s*, 3H-27), 1.00 (*s,* 3H-30), 1.17 (*s*, 3H-28) and 0.89 (*s*, 3H-29) ppm. Based on the NMR data and by comparison to literature values as shown in Tables S1 and S2, compound (3) was identified to be 3*β*-friedelanol (3) [23, 24].

Germanicol, (4) was isolated from the CH_2_Cl_2_ root extract of *E. grandicornis* as a white powder. The ^1^H NMR (400 MHz) spectrum presented singlets proton resonances due to eight methyl signals at δ_H_ (ppm); 0.78 (3H-24), 0.78 (3H-30), 0.82 (3H-28), 0.86 (3H-29), 1.00 (3H-23), 0.78 (3H-25), 0.95 (3H-26), 1.01 (3H-27), this was further confirmed by ^13^C NMR spectrum. In addition, it displayed proton signals between δ_H_ 1.12–2.01 ppm attributed to methine and methylene proton signals. The NMR spectral data and literature values as shown in Tables S1 and S2 confirmed the structure of the compound as germanicol [25–27].

### Identified compounds using LC-MS/MS

**Fig. 2:**
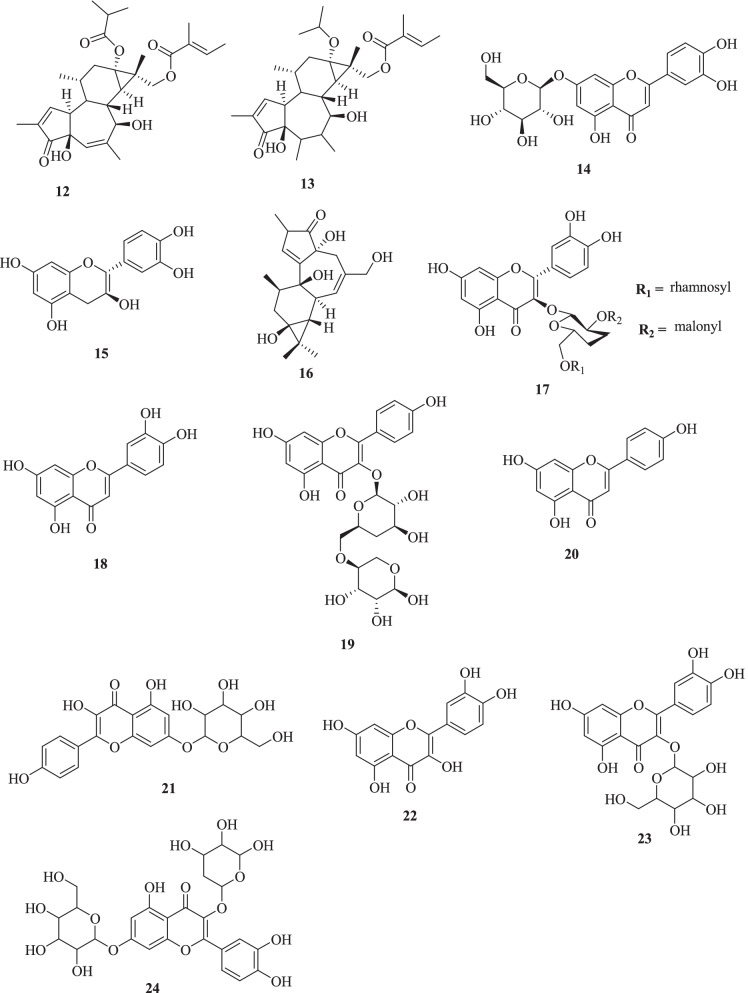
Structures of identified compounds from *Euphorbia grandicornis* Blanc crude extracts by LC-MS/MS

In this study, the phenolic profiling of CH_2_Cl_2_ aerial and root crude extracts of *E. grandicornis* was analyzed using LC-MS/MS in the positive and negative modes. The identification of compounds was based on the accurate comparison of MS profiles with the available reference standard, accurate mass, MS/MS fragmentation patterns, relative retention times, and by comparison with the literature data. Flavonoids **(14**-**24)** were detected as major components in the analyzed aerial extract of *E. grandicornis*. Diterpenes of phorbol esters; 16-angeloyloxy-13*R*-isobutanoyloxy-4*β*,9*R*,7*β*-trihydroxytiglia-1,5-dien-3-one **(12)**, 16-angeloyloxy-13*α*-isobutanoyloxy-4*β*,9*α*-dihydroxytiglia-1,6-dien-3-one **(13)** and ingenol **(16)**, were also identified. The identified flavonoids include apegenin **(14)**, catechin **(15)**, kaempferol 3-O-(2″-*O*-*α*-rhamnosyl-6″-*O*-malonyl)-*β*-glucoside **(17)**, kaempferol-*O*-glucosides **(18)**, kaempferol-*O*-rutinoside **(19)**, luteolin-*O*-glucoside **(20)**, luteolin **(21)**, quercetin **(22)**, quercetin-*O*-hexoside **(23)**, and rumarin (quercetin-*O*-pento hexoside) **(24)**, (Table 1, Fig. 2). Quercetin, quercetin-3-*O*-*α*-L-rhamnoside, and kaempferol-3-*O*-*α*-L-rhamnoside were previously isolated from *E. formosana* and apigenin-7-glucoside from *E. prostrata* [28].

**Table 1:** LC-MS/MS spectral data of compounds identified from crude extracts of *Euphorbia grandicornis* Blanc

No	Compound name	[M + H]^+^ or [M-H]^+^	MS fragmentation pattern (m/z)	Retention time (min)	Molecular Formula	Reference
**12**	16-angeloyloxy-13*R*-isobutanoyloxy-4*β*,9*R*,7*β*-trihydroxytiglia-1,5-dien-3-one	515.1188	312.9980 > 297.9784 > 269.9856	16.25	C_29_H_40_O_8_	[29]
**13**	16-angeloyloxy-13*α*-isobutanoyloxy-4*β*,9*α*-dihydroxytiglia-1,6-dien-3-one	499.9930	312.9980 > 297.9784 > 269.9856	18.03	C_29_H_40_O_7_	[29]
**14**	Apigenin	269.9856	225.9982 > 116.9274 > 151.0015	6.52	C_15_H_10_O_5_	[30]
**15**	Catechin	289.0690	230.1216 > 279.1215	17.13	C_15_H_12_O_6_	[29]
**16**	Ingenol	383.1900	312.9980 > 297.9784 > 269.9856	16.52	C_20_H_28_O_5_	[29]
**17**	Kaempferol 3-*O*-(2″-*O*-*α*-rhamnosyl-6″-*O*-malonyl)-*β*-glucoside	697.1594	343.0457, 301.0325, 284.0309 > 256.0275 > 226.0314	16.26	C_30_H_32_O_19_	[29, 31]
**18**	Kaempferol-*O*-glucoside	447.0910	284.0309 > 256.0275 > 226.0314	16.23	C_21_H_20_O_11_	[29]
**19**	Kaempferol-*O*-rutinoside	593.1503	284.0309 > 256.0275 > 226.0314	18.08	C_27_H_30_O_15_	[29, 32]
**20**	Luteolin-*O*-glucoside	449.2012	241.1439, 268.0359, 211.0379	16.18	C_21_H_20_O_11_	[33, 34]
**21**	Luteolin	285.0367	151.0031 > 125.0216	16.18	C_15_H_10_O_6_	[34]
**22**	Quercetin	301.0643	284.0275, 266.0388, 151.0011	16.22	C_15_H_10_O_7_	[35]
**23**	Quercetin-*O*-hexoside	463.1227	342.8297, 373.1298, 284.0275, 266.0388	16.25	C_21_H_20_O_12_	[35, 36]
**24**	Rumarin (quercetin-*O*-pento hexoside)	594.1510	464.2134, 373.1298, 284.0275, 266.0388	16.23	C_25_H_26_O_15_	[35]

The LC-MS chromatogram displayed a major peak [M-H]^+^ at *m/z* 593.1503 with a retention time of 18.08 min [Supplementary material (**LC-MS2**)]. These values were consistent with the theoretical molecular mass of kaempferol-*O*-rutinoside (**19**) and hence the peak was assigned to the known compound [29, 32]. The fragmentation patterns [M + H]^+^ observed at *m/z* 284.0309 (attributed to the loss of CH_3_)_,_ 285.0367, and 227.0314 were in agreement with that of the standard reference. Besides, the molecular ion peak [M-H]^−^ at *m/z* 447.0910 [Supplementary Material (**LC-MS3**)] with an MS fragmentation ion at *m/z* 285.0367 and which was consistent with the reference standard, was assigned as kaempferol-*O*-glucoside (**18**) [29]. These flavonoids were the most abundant phenolic compounds in the aerial crude extract.

The peak [M-H]^−^ *at m/z* 463.1227 [Supplementary Material (**LC-MS4**)] was assigned as quercetin-*O*-hexoside (**23**). The LC-MS spectral data of quercetin-*O*-hexoside agreed with the previously reported quercetin hexoside by Hussein et al. [36]. Moreover, a molecular ion peak [M-H]^−^ at *m/z* 289.0690 with a fragmentation molecular ion at *m/z* 230.1216 and *m/z* 279.1215 and retention time 17.13 min [Supplementary Material (**LC-MS6**)] was assigned as (+)-catechin (**15**) previously identified in *Argan* fruits. The fragmentation pattern was observed, and its retention time was comparable with the reference standard and with the theoretical data for catechin. The observed peak [M + H]^+^ at *m/z* 449.2012 and the fragmentation patterns [Supplementary Material (LC-MS6)] helped to assign this peak to luteolin-*O*-glucoside (**20**), previously isolated from thyme by [33] and identified from *Chrysanthemum* flower. The MS data were consistent with the reported compound.

In addition, a major molecular peak [M + H]^+^ at *m/z* 697.1594 (for C_30_H_32_O_19_) with a retention time of 16.26 min [Supplementary material (**LC-MS5**)] was attributed to a flavonoid glycoside kaempferol 3-*O*-(2″-*O*-*α*-rhamnosyl-6″-*O*-malonyl)-*β*-glucoside (**17**), a rhamnosyl flavonoid previously isolated from *Clitoria termatea* [31]. The fragmentation pattern for this compound at *m/z* 301.0325 was consistent with the theoretical MS data for the compound.

### Antibacterial activities isolated compounds

This study revealed that the CH_2_Cl_2_ extract had antibacterial activities against *Escherichia coli* ATCC 8739 and *Staphylococcus aureus* ATCC 6538 with a percentage growth of 94.90 ± 4.24 and 29.47 ± 4.89 μg/mL respectively. Table 2 shows the percentage growth of *S. aureus*, *E. coli,* and HeLa cells on exposure to crude extracts and pure compounds.

**Table 2:** Percentage growth of *S. aureus*, *E. coli,* and HeLa cells on exposure to 50 μg/mL of the isolated compounds

No	Compound name	*E. coli*	*S. aureus*	HeLa cells
**1**	Methyl 2,5-dihydroxybenzoate	19.12 ± 0.65	23.32 ± 0.23	121.06 ± 3.45
**2**	*n-*octylbenzoate	106.52 ± 7.55	76.07 ± 1.94	105.49 ± 2.52
**3**	Friedelanol	123.95 ± 4.06	66.99 ± 5.82	113.41 ± 3.11
**4**	Germanicol	112.06 ± 3.30	71.93 ± 9.58	130.01 ± 1.10
**5**	*β-*glutinol	116.17 ± 1.02	78.84 ± 21.64	128.04 ± 5.92
**6**	*β-*amyrin	90.86 ± 13.91	27.17 ± 0.07	102.89 ± 15.54
**7**	Stigmasterol	93.58 ± 5.72	87.89 ± 7.36	15.24 ± 0.26
**8**	*β-*Sitosterol	102.21 ± 3.67	47.79 ± 2.99	119.24 ± 11.34
**9**	(24*R*)-tirucalla-8,25-diene-3*β*, 24-diol	103.27 ± 2.27	51.94 ± 15.59	18.81 ± 2.02
**10**	Euphorbol	113.93 ± 9.14	70.21 ± 2.31	50.57 ± 11.29
**11**	Hexyl (*E*)-3-(4-hydroxy-3-methoxyphenyl)-2-propenoate	110.53 ± 1.08	83.16 ± 8.67	117.39 ± 9.71
Crude	CH_2_Cl_2_ aerial extracts	94.90 ± 4.24	29.47 ± 4.89	83.84 ± 2.94

Negative control 0% media only, no cells, and positive control 100% cells plus media (no extract) were used

The extract was more active against *S. aureus* indicating selective activity with a percentage growth of 94.90 ± 4.24 and 29.47 ± 4.89 against *E. coli* and *S. aureus* respectively. The results are in agreement with the previous studies which showed significant antibacterial activities of *E. royleana* hexane extracts [37]. The extract exhibited antibacterial activity against the Gram-positive bacteria *Bacillus subtilis* [38]. Antibacterial evaluation of extracts from *E. guyoniana* showed significant activities against strains used with percentage growth values ranging from 1.47 to 61.78 mg/mL in the order of *Staphylococcus aureus* > *Streptococcus faecalis* > *Escherichia coli* [38].

**Fig. 3:**
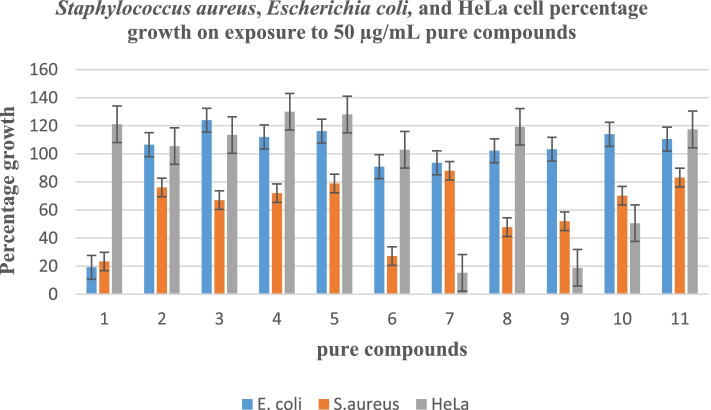
Percentage growth of *S. aureus*, *E. coli* and Hela cells

Among the tested compounds, Methyl 2,5-dihydroxybenzoate (**1**), was the most active against *E.coli* and *S. aureus* with percentage growth values of 19.12 ± 0.65 and 23.32 ± 0.23 μg/mL respectively. *β*-amyrin (**6**), and *β*-sitosterol (**8**), were active against *S. aureus* with percentage growth values of 27.17 ± 0.07, and 47.79 ± 2.99 μg/mL respectively. Other compounds exhibited some antibacterial activities with percentage growth values ranging from 51.94 ± 15.59 to 83.57 ± 4.45 for *S. aureus* and from 79.10 ± 1.36 to 132.55 ± 2.16 for *E. coli* as presented in Table 2 and Fig. 3.

It was interesting to note that stigmasterol (**7**), (24*R*)-tirucalla-8,25-diene-3*β*, 24-diol (**9**), and euphorbol (**10**) exhibited cytotoxic activities against HeLa cells with values of 15.24 ± 0.26, 18.81 ± 2.02 and 50.57 ± 11.29 respectively, as illustrated in Fig. 3.

In related studies, evaluation of compounds isolated from *E. tirucalli* exhibited significant antibacterial activities of the extracts against *S. aureus* (ATCC 6538), *S. brasiliensis* (UFPE 121), and *E. coli* (ATCC 8739), with minimum inhibition concentration (MIC) values ranging from 256 to 1024 μg/mL. Of the tested compounds, ampelopsin showed a MIC value of 16 μg/mL against *E. coli* (ATCC 8739), compared to tetracycline (MIC value of 32 μg/mL) [39]. Equally, the evaluation of hexane extract of *E. royleana* displayed significant antimicrobial activities [37].

## Conclusions

Chromatographic analysis of *Euphorbia grandicornis* Blanc crude extracts afforded twenty-four compounds (**1**-**24**). Compounds (**1**-**4**) were reported for the first time from the species. Methyl 2,5-dihydroxybenzoate (**1**) displayed good antibacterial activities against *E. coli* and *S. aureus* with a percentage growth of 19.12 and 23.32 at 50 μg/mL respectively. The results obtained from this study shows that *E. grandicornis,* is rich source of chemical constituents that may provide new lead compounds for the development of antibacterial agents.

## Supplementary Information

**Additional file 1: Supplementary Materials.** The following are available online alongside Appendix 1–12, Appendix LC-MS1-LCMS6, **Table S1.** ^13^C (δc) (100.6 MHZ) NMR data for compounds **1**-**11** in CDCl3, **Table S2.** ^1^H (δH, m, (*J*, Hz) NMR (400 MHZ) data for compounds 1–4 in CDCl_3_.

## Data Availability

All data generated or analysed during this study are included in this published article [and its supplementary information files].
